# 2-(1-Propyl-2,6-distyryl-1,4-dihydro­pyridin-4-yl­idene)malononitrile

**DOI:** 10.1107/S1600536809048892

**Published:** 2009-11-21

**Authors:** Kwang Ha, Joon Heo, Hyung Jin Kim

**Affiliations:** a School of Applied Chemical Engineering, Center for Functional Nano Fine Chemicals, Chonnam National University, Gwangju 500-757, Republic of Korea

## Abstract

In the title compound, C_27_H_23_N_3_, the dihedral angles between the central pyridine ring and the two outer benzene rings are 32.6 (1) and 52.0 (1)°. The compound displays inter­molecular π–π inter­actions between adjacent six-membered rings, the shortest centroid–centroid distance being 3.981 (3) Å.

## Related literature

For the synthesis of the starting material, 2-(2,6-dimethyl­pyridin-4(1*H*)-yl­idene)malononitrile, see: Kato *et al.* (1960 ▶). For an alternative synthesis of the title compound, see: Peng *et al.* (2006 ▶). For the uses and crystal structures of 4-(dicyano­methyl­ene)-2-methyl-6-[(dimethyl­amino)styr­yl]-4*H*-pyran derivatives, see: Tang *et al.* (1989 ▶); Chen *et al.* (2000 ▶); Ju *et al.* (2006 ▶); Tong *et al.* (2006 ▶).
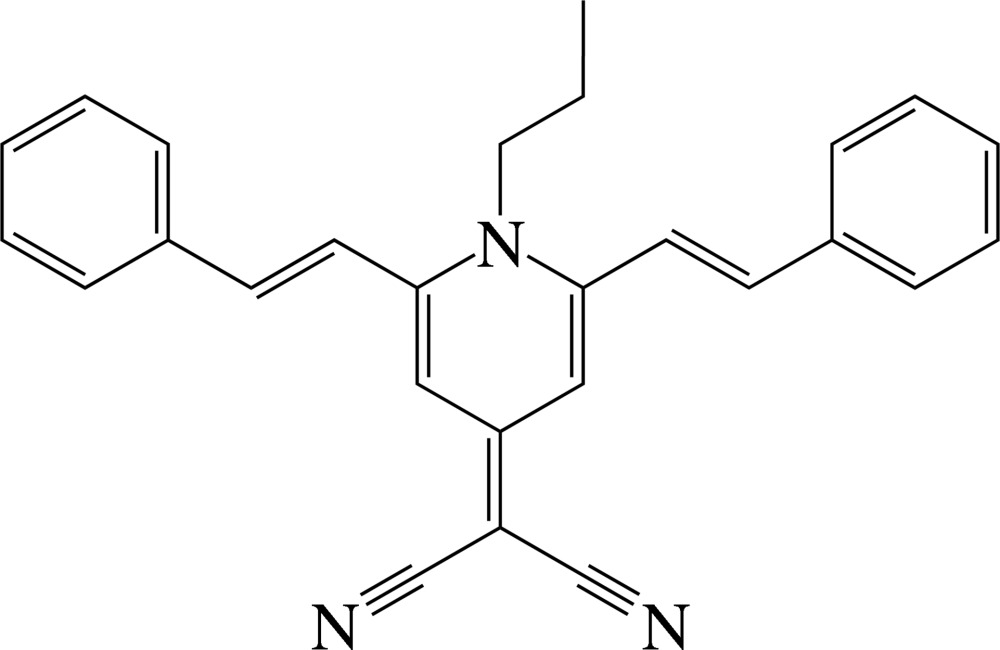


## Experimental

### 

#### Crystal data

C_27_H_23_N_3_
*M* *_r_* = 389.48
Monoclinic, 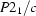

*a* = 16.586 (2) Å
*b* = 17.827 (2) Å
*c* = 7.4543 (9) Å
β = 99.790 (3)°
*V* = 2171.9 (5) Å^3^
*Z* = 4
Mo *K*α radiation
μ = 0.07 mm^−1^
*T* = 293 K
0.20 × 0.20 × 0.17 mm

#### Data collection

Bruker SMART 1000 CCD diffractometer
Absorption correction: multi-scan (*SADABS*; Bruker, 2000 ▶) *T*_min_ = 0.630, *T*_max_ = 1.000
12600 measured reflections
4433 independent reflections
2084 reflections with *I* > 2σ(*I*)
*R*_int_ = 0.033

#### Refinement

*R*[*F* ^2^ > 2σ(*F* ^2^)] = 0.061
*wR*(*F* ^2^) = 0.201
*S* = 1.01
4433 reflections
272 parameters
H-atom parameters constrained
Δρ_max_ = 0.14 e Å^−3^
Δρ_min_ = −0.16 e Å^−3^

### 

Data collection: *SMART* (Bruker, 2000 ▶); cell refinement: *SAINT* (Bruker, 2000 ▶); data reduction: *SAINT*; program(s) used to solve structure: *SHELXS97* (Sheldrick, 2008 ▶); program(s) used to refine structure: *SHELXL97* (Sheldrick, 2008 ▶); molecular graphics: *ORTEP-3* (Farrugia, 1997 ▶) and *PLATON* (Spek, 2009 ▶); software used to prepare material for publication: *SHELXL97*.

## Supplementary Material

Crystal structure: contains datablocks global, I. DOI: 10.1107/S1600536809048892/ng2689sup1.cif

Structure factors: contains datablocks I. DOI: 10.1107/S1600536809048892/ng2689Isup2.hkl

Additional supplementary materials:  crystallographic information; 3D view; checkCIF report

## Figures and Tables

**Fig. 1.**
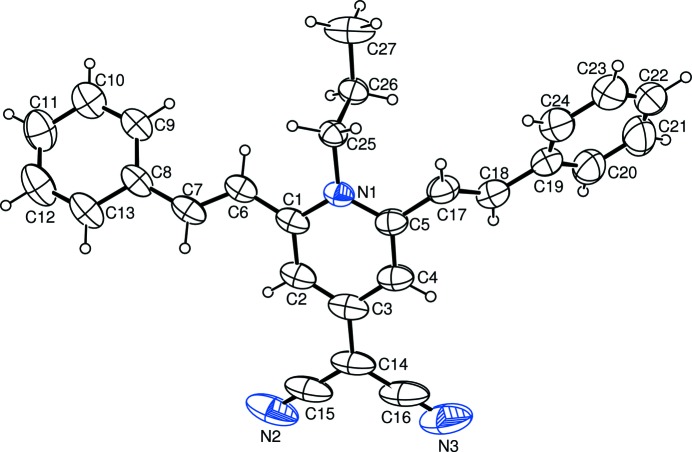
The structure of the title compound, with displacement ellipsoids drawn at the 30% probability level for non-H atoms.

**Fig. 2.**
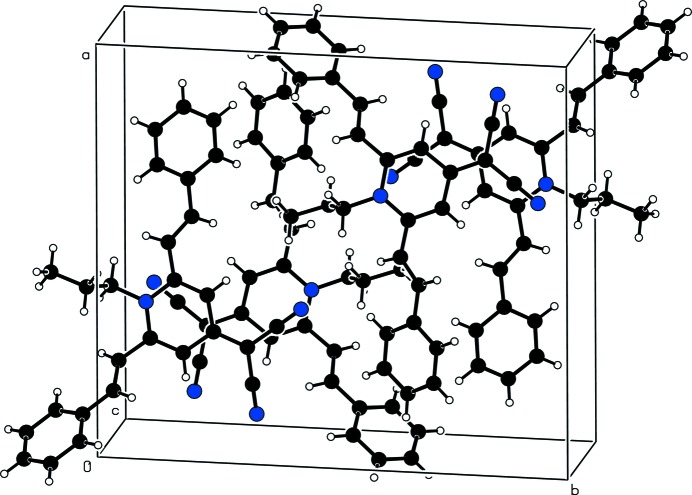
View of the unit-cell contents of the title compound.

## supplementary crystallographic information

### Comment

4-(Dicyanomethylene)-2-methyl-6-[(dimethylamino)styryl]-4*H*-pyran (DCM) and its analogs have been utilized as highly fluorescent dopants in organic light-emitting diodes (OLED) (Tang *et al.*, 1989; Chen *et al.*, 2000). In a recent year, a modified DCM, 2-[2,6-bis(2-arylvinyl)pyridin-4-ylidene]malononitrile (BPM), has been prepared from the corresponding (pyran-4-yliden)malononitrile derivative to obtain an efficient fluorescent material for OLED (Peng *et al.*, 2006). However, their yields were low. To improve the yields of BPM derivatives, we synthesized the title compound by Knoevenagel condensation of 2-(2,6-dimethyl-1-propylpyridin-4(1*H*)-ylidene)malononitrile with benzaldehyde and characterized its structure.

In the title compound, C_27_H_23_N_3_, the dihedral angles between the central pyridine ring and the two outer benzene rings are 32.6 (1)° and 52.0 (1)°, respectively, and the dihedral angle between the benzene rings is 74.3 (1)° (Fig. 1). The dicyanomethylene group lies in the pyridine ring plane with the largest deviation of 0.212 (7) Å (N3) from the least-squares plane of the pyridine ring. The compound displays intermolecular π-π interactions between the adjacent six-membered rings (the symmetry operation for second plane -*x*,-*y*,-*z*), with a shortest centroid-centroid distance of 3.981 (3) Å, and the planes are parallel and shifted for 1.926 Å (Fig. 2).

### Experimental

A mixture of 2-(2,6-dimethyl-1-propylpyridin-4(1*H*)-ylidene)malononitrile (1.015 g, 4.76 mmol), benzaldehyde (1.111 g, 10.47 mmol) and piperidine (0.5 ml) in DMF (10 ml) was stirred and heated for 12 h at 100 °C under nitrogen. After cooling to room temperature the mixture was concentrated under vacuum to give the crude product, which was column chromatographed (SiO_2_) by eluting with a mixture of acetone/CHCl_3_ (1:20) to afford the title compound (1.290 g, 70%) as an orange solid. Crystals suitable for X-ray analysis were obtained by slow evaporation from a CHCl_3_/EtOH solution. Mp 252–253 °C. ^1^H NMR (300 MHz, CDCl_3_): δ 7.55–7.40 (m, 10H, Ph), 7.23 (d, 2H, J = 15.6 Hz, –CH=C*H*-Ph), 7.01 (s, 2H, C*H*=C of pyridine), 6.96 (d, 2H, J = 15.6 Hz, –C*H*=CH—Ph), 4.03 (t, 2H, J = 8.1 Hz, NC*H*_2_CH_2_), 1.87 (m, 2H, –CH_2_C*H*_2_CH_3_), 1.02 (t, 3H, J = 7.2 Hz, –CH_2_C*H*_3_). ^13^C NMR (75 MHz, CDCl_3_): δ 155.8, 148.0, 139.8, 134.8, 130.0, 129.1, 127.5, 118.7, 111.7, 51.4, 47.1, 23.3, 11.0.

### Refinement

H atoms were positioned geometrically and allowed to ride on their respective parent atoms [C—H = 0.93 (CH), 0.97 (CH_2_) or 0.96 Å (CH_3_) and *U*_iso_(H) = 1.2*U*_eq_ or 1.5*U*_eq_(methyl C)].

### Crystal data

**Table:**

C_27_H_23_N_3_	*F*(000) = 824
*M**_r_* = 389.48	*D*_x_ = 1.191 Mg m^−^^3^
Monoclinic, *P*2_1_/*c*	Mo *K*α radiation, λ = 0.71073 Å
Hall symbol: -P 2ybc	Cell parameters from 690 reflections
*a* = 16.586 (2) Å	θ = 2.5–19.1°
*b* = 17.827 (2) Å	µ = 0.07 mm^−^^1^
*c* = 7.4543 (9) Å	*T* = 293 K
β = 99.790 (3)°	Stick, orange
*V* = 2171.9 (5) Å^3^	0.20 × 0.20 × 0.17 mm
*Z* = 4	

### Data collection

**Table:**

Bruker SMART 1000 CCD diffractometer	4433 independent reflections
Radiation source: fine-focus sealed tube	2084 reflections with *I* > 2σ(*I*)
graphite	*R*_int_ = 0.033
φ and ω scans	θ_max_ = 26.4°, θ_min_ = 2.3°
Absorption correction: multi-scan (*SADABS*; Bruker, 2000)	*h* = −17→20
*T*_min_ = 0.630, *T*_max_ = 1.000	*k* = −22→22
12600 measured reflections	*l* = −9→9

### Refinement

**Table:**

Refinement on *F*^2^	Primary atom site location: structure-invariant direct methods
Least-squares matrix: full	Secondary atom site location: difference Fourier map
*R*[*F*^2^ > 2σ(*F*^2^)] = 0.061	Hydrogen site location: inferred from neighbouring sites
*wR*(*F*^2^) = 0.201	H-atom parameters constrained
*S* = 1.01	*w* = 1/[σ^2^(*F*_o_^2^) + (0.0808*P*)^2^ + 0.1288*P*] where *P* = (*F*_o_^2^ + 2*F*_c_^2^)/3
4433 reflections	(Δ/σ)_max_ < 0.001
272 parameters	Δρ_max_ = 0.14 e Å^−^^3^
0 restraints	Δρ_min_ = −0.16 e Å^−^^3^

### Special details

**Table:**

Geometry. All e.s.d.'s (except the e.s.d. in the dihedral angle between two l.s. planes) are estimated using the full covariance matrix. The cell e.s.d.'s are taken into account individually in the estimation of e.s.d.'s in distances, angles and torsion angles; correlations between e.s.d.'s in cell parameters are only used when they are defined by crystal symmetry. An approximate (isotropic) treatment of cell e.s.d.'s is used for estimating e.s.d.'s involving l.s. planes.
Refinement. Refinement of *F*^2^ against ALL reflections. The weighted *R*-factor *wR* and goodness of fit *S* are based on *F*^2^, conventional *R*-factors *R* are based on *F*, with *F* set to zero for negative *F*^2^. The threshold expression of *F*^2^ > σ(*F*^2^) is used only for calculating *R*-factors(gt) *etc*. and is not relevant to the choice of reflections for refinement. *R*-factors based on *F*^2^ are statistically about twice as large as those based on *F*, and *R*- factors based on ALL data will be even larger.

### Fractional atomic coordinates and isotropic or equivalent isotropic displacement parameters (Å^2^)

**Table:**

	*x*	*y*	*z*	*U*_iso_*/*U*_eq_	
N1	0.35774 (14)	0.08906 (10)	0.2536 (3)	0.0713 (6)	
N2	0.3616 (3)	0.42152 (17)	0.2101 (5)	0.1696 (17)	
N3	0.1110 (3)	0.3314 (2)	0.1271 (6)	0.186 (2)	
C1	0.4116 (2)	0.14869 (13)	0.2691 (3)	0.0772 (8)	
C2	0.3815 (2)	0.21977 (15)	0.2438 (4)	0.0889 (9)	
H2	0.4182	0.2596	0.2518	0.107*	
C3	0.2971 (3)	0.23522 (16)	0.2060 (4)	0.0938 (10)	
C4	0.2452 (2)	0.17187 (15)	0.1836 (4)	0.0900 (9)	
H4	0.1889	0.1791	0.1539	0.108*	
C5	0.27456 (19)	0.10081 (14)	0.2040 (4)	0.0780 (7)	
C6	0.49940 (19)	0.13383 (15)	0.3018 (4)	0.0803 (8)	
H6	0.5171	0.0897	0.2545	0.096*	
C7	0.5559 (2)	0.17842 (14)	0.3937 (4)	0.0843 (8)	
H7	0.5369	0.2203	0.4483	0.101*	
C8	0.6446 (2)	0.16912 (15)	0.4189 (4)	0.0828 (8)	
C9	0.6811 (2)	0.10774 (16)	0.3513 (4)	0.0902 (9)	
H9	0.6487	0.0697	0.2918	0.108*	
C10	0.7643 (3)	0.1027 (2)	0.3714 (5)	0.1122 (11)	
H10	0.7879	0.0614	0.3243	0.135*	
C11	0.8137 (2)	0.1581 (2)	0.4608 (5)	0.1264 (13)	
H11	0.8703	0.1547	0.4722	0.152*	
C12	0.7787 (3)	0.2177 (2)	0.5321 (5)	0.1301 (14)	
H12	0.8116	0.2549	0.5942	0.156*	
C13	0.6951 (2)	0.22324 (18)	0.5128 (4)	0.1046 (11)	
H13	0.6720	0.2639	0.5635	0.125*	
C14	0.2655 (3)	0.30813 (17)	0.1869 (4)	0.1153 (13)	
C15	0.3178 (3)	0.3705 (2)	0.2000 (5)	0.1326 (16)	
C16	0.1804 (4)	0.3210 (2)	0.1529 (6)	0.143 (2)	
C17	0.22033 (18)	0.03589 (15)	0.1665 (4)	0.0869 (8)	
H17	0.2284	−0.0049	0.2451	0.104*	
C18	0.1602 (2)	0.03298 (17)	0.0248 (5)	0.0929 (9)	
H18	0.1523	0.0759	−0.0471	0.111*	
C19	0.1048 (2)	−0.0295 (2)	−0.0320 (6)	0.1017 (10)	
C20	0.0541 (2)	−0.0257 (2)	−0.1978 (6)	0.1274 (13)	
H20	0.0540	0.0172	−0.2686	0.153*	
C21	0.0036 (3)	−0.0844 (3)	−0.2604 (8)	0.166 (2)	
H21	−0.0296	−0.0811	−0.3740	0.199*	
C22	0.0015 (3)	−0.1471 (3)	−0.1589 (12)	0.167 (3)	
H22	−0.0327	−0.1867	−0.2026	0.200*	
C23	0.0501 (3)	−0.1516 (3)	0.0085 (10)	0.171 (2)	
H23	0.0485	−0.1942	0.0800	0.206*	
C24	0.1018 (2)	−0.0927 (2)	0.0712 (7)	0.1312 (13)	
H24	0.1348	−0.0960	0.1849	0.157*	
C25	0.38929 (15)	0.01206 (12)	0.2886 (3)	0.0690 (7)	
H25A	0.3520	−0.0159	0.3506	0.083*	
H25B	0.4418	0.0142	0.3687	0.083*	
C26	0.39921 (16)	−0.02907 (13)	0.1163 (3)	0.0744 (7)	
H26A	0.3472	−0.0299	0.0341	0.089*	
H26B	0.4382	−0.0024	0.0565	0.089*	
C27	0.4281 (3)	−0.10736 (17)	0.1544 (5)	0.1335 (14)	
H27A	0.4785	−0.1069	0.2395	0.200*	
H27B	0.4368	−0.1307	0.0432	0.200*	
H27C	0.3876	−0.1350	0.2049	0.200*	

### Atomic displacement parameters (Å^2^)

**Table:**

	*U*^11^	*U*^22^	*U*^33^	*U*^12^	*U*^13^	*U*^23^
N1	0.0922 (16)	0.0552 (12)	0.0665 (13)	0.0047 (11)	0.0136 (11)	0.0026 (10)
N2	0.326 (5)	0.0653 (18)	0.119 (3)	0.013 (3)	0.043 (3)	0.0021 (19)
N3	0.238 (5)	0.138 (3)	0.175 (4)	0.105 (3)	0.018 (3)	0.009 (2)
C1	0.118 (2)	0.0539 (15)	0.0615 (16)	−0.0031 (15)	0.0220 (15)	−0.0006 (12)
C2	0.137 (3)	0.0596 (17)	0.0723 (18)	0.0013 (17)	0.0248 (18)	−0.0003 (13)
C3	0.164 (3)	0.0635 (18)	0.0571 (16)	0.023 (2)	0.0264 (18)	0.0057 (13)
C4	0.123 (2)	0.0749 (19)	0.0732 (18)	0.0276 (17)	0.0181 (16)	0.0061 (14)
C5	0.095 (2)	0.0701 (17)	0.0693 (17)	0.0147 (15)	0.0158 (15)	0.0058 (13)
C6	0.109 (2)	0.0616 (16)	0.0746 (17)	−0.0105 (16)	0.0265 (16)	−0.0036 (14)
C7	0.131 (3)	0.0583 (16)	0.0676 (17)	−0.0189 (16)	0.0293 (17)	−0.0045 (13)
C8	0.117 (3)	0.0737 (18)	0.0625 (16)	−0.0325 (17)	0.0299 (16)	−0.0055 (13)
C9	0.114 (3)	0.083 (2)	0.0746 (19)	−0.0237 (17)	0.0218 (18)	−0.0060 (15)
C10	0.124 (3)	0.122 (3)	0.096 (2)	−0.021 (2)	0.032 (2)	−0.010 (2)
C11	0.115 (3)	0.159 (4)	0.110 (3)	−0.042 (3)	0.033 (2)	−0.014 (3)
C12	0.149 (4)	0.143 (4)	0.106 (3)	−0.068 (3)	0.040 (3)	−0.028 (2)
C13	0.138 (3)	0.095 (2)	0.087 (2)	−0.047 (2)	0.037 (2)	−0.0206 (17)
C14	0.202 (4)	0.064 (2)	0.079 (2)	0.044 (2)	0.021 (2)	0.0034 (16)
C15	0.254 (5)	0.062 (2)	0.084 (2)	0.039 (3)	0.032 (3)	0.0040 (19)
C16	0.237 (6)	0.086 (3)	0.103 (3)	0.077 (3)	0.021 (4)	0.0076 (19)
C17	0.086 (2)	0.0750 (19)	0.101 (2)	0.0131 (15)	0.0182 (18)	0.0124 (16)
C18	0.092 (2)	0.088 (2)	0.099 (2)	0.0194 (17)	0.0166 (19)	0.0030 (17)
C19	0.080 (2)	0.095 (3)	0.131 (3)	0.0138 (18)	0.022 (2)	−0.020 (2)
C20	0.098 (3)	0.147 (4)	0.136 (3)	0.009 (2)	0.014 (3)	−0.037 (3)
C21	0.106 (3)	0.189 (5)	0.196 (6)	−0.006 (4)	0.012 (3)	−0.087 (5)
C22	0.086 (3)	0.123 (4)	0.298 (9)	−0.004 (3)	0.049 (4)	−0.076 (5)
C23	0.102 (4)	0.111 (4)	0.302 (8)	0.000 (3)	0.036 (4)	−0.021 (4)
C24	0.102 (3)	0.099 (3)	0.192 (4)	0.001 (2)	0.023 (3)	−0.001 (3)
C25	0.0808 (17)	0.0565 (14)	0.0680 (16)	−0.0001 (11)	0.0082 (13)	0.0074 (12)
C26	0.0885 (18)	0.0652 (15)	0.0711 (16)	−0.0002 (13)	0.0181 (14)	0.0013 (12)
C27	0.231 (4)	0.079 (2)	0.102 (3)	0.049 (2)	0.062 (3)	0.0116 (18)

### Geometric parameters (Å, °)

**Table:**

N1—C1	1.381 (3)	C13—H13	0.9300
N1—C5	1.382 (3)	C14—C15	1.402 (6)
N1—C25	1.476 (3)	C14—C16	1.411 (6)
N2—C15	1.160 (5)	C17—C18	1.324 (4)
N3—C16	1.148 (6)	C17—H17	0.9300
C1—C2	1.363 (4)	C18—C19	1.460 (4)
C1—C6	1.459 (4)	C18—H18	0.9300
C2—C3	1.408 (4)	C19—C24	1.369 (5)
C2—H2	0.9300	C19—C20	1.373 (5)
C3—C14	1.399 (4)	C20—C21	1.372 (5)
C3—C4	1.413 (4)	C20—H20	0.9300
C4—C5	1.357 (3)	C21—C22	1.353 (7)
C4—H4	0.9300	C21—H21	0.9300
C5—C17	1.463 (4)	C22—C23	1.367 (8)
C6—C7	1.327 (4)	C22—H22	0.9300
C6—H6	0.9300	C23—C24	1.386 (6)
C7—C8	1.461 (4)	C23—H23	0.9300
C7—H7	0.9300	C24—H24	0.9300
C8—C9	1.386 (4)	C25—C26	1.512 (3)
C8—C13	1.387 (4)	C25—H25A	0.9700
C9—C10	1.364 (4)	C25—H25B	0.9700
C9—H9	0.9300	C26—C27	1.487 (3)
C10—C11	1.380 (4)	C26—H26A	0.9700
C10—H10	0.9300	C26—H26B	0.9700
C11—C12	1.362 (5)	C27—H27A	0.9600
C11—H11	0.9300	C27—H27B	0.9600
C12—C13	1.373 (5)	C27—H27C	0.9600
C12—H12	0.9300		
			
C1—N1—C5	120.5 (2)	N2—C15—C14	179.2 (5)
C1—N1—C25	119.7 (2)	N3—C16—C14	179.3 (5)
C5—N1—C25	119.7 (2)	C18—C17—C5	122.6 (3)
C2—C1—N1	119.1 (3)	C18—C17—H17	118.7
C2—C1—C6	121.6 (3)	C5—C17—H17	118.7
N1—C1—C6	119.2 (2)	C17—C18—C19	127.8 (3)
C1—C2—C3	122.6 (3)	C17—C18—H18	116.1
C1—C2—H2	118.7	C19—C18—H18	116.1
C3—C2—H2	118.7	C24—C19—C20	118.1 (4)
C14—C3—C2	123.0 (4)	C24—C19—C18	123.1 (4)
C14—C3—C4	121.4 (4)	C20—C19—C18	118.8 (4)
C2—C3—C4	115.6 (3)	C21—C20—C19	120.9 (5)
C5—C4—C3	122.2 (3)	C21—C20—H20	119.5
C5—C4—H4	118.9	C19—C20—H20	119.5
C3—C4—H4	118.9	C22—C21—C20	120.7 (6)
C4—C5—N1	119.7 (3)	C22—C21—H21	119.6
C4—C5—C17	121.3 (3)	C20—C21—H21	119.6
N1—C5—C17	119.0 (2)	C21—C22—C23	119.5 (5)
C7—C6—C1	125.1 (3)	C21—C22—H22	120.2
C7—C6—H6	117.5	C23—C22—H22	120.2
C1—C6—H6	117.5	C22—C23—C24	119.8 (6)
C6—C7—C8	127.3 (3)	C22—C23—H23	120.1
C6—C7—H7	116.4	C24—C23—H23	120.1
C8—C7—H7	116.4	C19—C24—C23	120.9 (5)
C9—C8—C13	118.0 (3)	C19—C24—H24	119.5
C9—C8—C7	122.4 (3)	C23—C24—H24	119.5
C13—C8—C7	119.6 (3)	N1—C25—C26	112.79 (19)
C10—C9—C8	120.5 (3)	N1—C25—H25A	109.0
C10—C9—H9	119.7	C26—C25—H25A	109.0
C8—C9—H9	119.7	N1—C25—H25B	109.0
C9—C10—C11	120.8 (4)	C26—C25—H25B	109.0
C9—C10—H10	119.6	H25A—C25—H25B	107.8
C11—C10—H10	119.6	C27—C26—C25	111.8 (2)
C12—C11—C10	119.3 (4)	C27—C26—H26A	109.3
C12—C11—H11	120.3	C25—C26—H26A	109.3
C10—C11—H11	120.3	C27—C26—H26B	109.3
C11—C12—C13	120.3 (3)	C25—C26—H26B	109.3
C11—C12—H12	119.9	H26A—C26—H26B	107.9
C13—C12—H12	119.9	C26—C27—H27A	109.5
C12—C13—C8	121.0 (3)	C26—C27—H27B	109.5
C12—C13—H13	119.5	H27A—C27—H27B	109.5
C8—C13—H13	119.5	C26—C27—H27C	109.5
C3—C14—C15	120.9 (4)	H27A—C27—H27C	109.5
C3—C14—C16	121.0 (4)	H27B—C27—H27C	109.5
C15—C14—C16	118.1 (3)		
			
C5—N1—C1—C2	3.2 (3)	C10—C11—C12—C13	−1.0 (6)
C25—N1—C1—C2	−177.3 (2)	C11—C12—C13—C8	−0.8 (5)
C5—N1—C1—C6	−173.3 (2)	C9—C8—C13—C12	2.5 (4)
C25—N1—C1—C6	6.2 (3)	C7—C8—C13—C12	−177.2 (3)
N1—C1—C2—C3	1.4 (4)	C2—C3—C14—C15	1.7 (4)
C6—C1—C2—C3	177.8 (2)	C4—C3—C14—C15	−176.7 (3)
C1—C2—C3—C14	177.4 (3)	C2—C3—C14—C16	−178.6 (3)
C1—C2—C3—C4	−4.1 (4)	C4—C3—C14—C16	3.0 (4)
C14—C3—C4—C5	−179.1 (3)	C4—C5—C17—C18	41.4 (4)
C2—C3—C4—C5	2.4 (4)	N1—C5—C17—C18	−135.8 (3)
C3—C4—C5—N1	2.0 (4)	C5—C17—C18—C19	176.8 (3)
C3—C4—C5—C17	−175.2 (3)	C17—C18—C19—C24	8.4 (5)
C1—N1—C5—C4	−4.9 (4)	C17—C18—C19—C20	−170.6 (3)
C25—N1—C5—C4	175.7 (2)	C24—C19—C20—C21	−1.8 (5)
C1—N1—C5—C17	172.3 (2)	C18—C19—C20—C21	177.2 (3)
C25—N1—C5—C17	−7.1 (3)	C19—C20—C21—C22	1.1 (7)
C2—C1—C6—C7	34.3 (4)	C20—C21—C22—C23	0.4 (8)
N1—C1—C6—C7	−149.3 (2)	C21—C22—C23—C24	−1.0 (8)
C1—C6—C7—C8	−175.1 (2)	C20—C19—C24—C23	1.2 (5)
C6—C7—C8—C9	−2.6 (4)	C18—C19—C24—C23	−177.8 (3)
C6—C7—C8—C13	177.1 (3)	C22—C23—C24—C19	0.2 (7)
C13—C8—C9—C10	−2.4 (4)	C1—N1—C25—C26	−96.4 (3)
C7—C8—C9—C10	177.3 (3)	C5—N1—C25—C26	83.0 (3)
C8—C9—C10—C11	0.6 (5)	N1—C25—C26—C27	−177.9 (3)
C9—C10—C11—C12	1.1 (6)		
